# Spatial Resolution and Refractive Index Contrast of Resonant Photonic Crystal Surfaces for Biosensing

**DOI:** 10.1109/JPHOT.2015.2435699

**Published:** 2015-06-01

**Authors:** G. J. Triggs, M. Fischer, D. Stellinga, M. G. Scullion, G. J. O. Evans, T. F. Krauss

**Affiliations:** 1 Department of Physics, University of York, York YO24 1UB, U.K.; 2 Department of Biology and Hull York Medical School, University of York, York, YO24 1UB, U.K.

**Keywords:** Resonant surface, biosensor, grating, photonic crystal, spatial resolution, refractive index contrast, polarization

## Abstract

By depositing a resolution test pattern on top of a Si_3_N_4_ photonic crystal resonant surface, we have measured the dependence of spatial resolution on refractive index contrast Δ*n*. Our experimental results and finite-difference time-domain (FDTD) simulations at different refractive index contrasts show that the spatial resolution of our device reduces with reduced contrast, which is an important consideration in biosensing, where the contrast may be of order 10^−2^. We also compare 1-D and 2-D gratings, taking into account different incidence polarizations, leading to a better understanding of the excitation and propagation of the resonant modes in these structures, as well as how this contributes to the spatial resolution. At Δ*n* = 0.077, we observe resolutions of 2 and 6 *μ*m parallel to and perpendicular to the grooves of a 1-D grating, respectively, and show that for polarized illumination of a 2-D grating, resolution remains asymmetrical. Illumination of a 2-D grating at 45° results in symmetric resolution. At very low index contrast, the resolution worsens dramatically, particularly for Δ*n* < 0.01, where we observe a resolution exceeding 10 *μ*m for our device. In addition, we measure a reduction in the resonance linewidth as the index contrast becomes lower, corresponding to a longer resonant mode propagation length in the structure and contributing to the change in spatial resolution.

## 1. Introduction

Photonic crystal biosensors based on resonant surfaces have been developed over the past decade for highly sensitive label-free sensing [1]–[7]. In line with other photonic sensors, a refractive index change at the surface causes a detectable shift of the resonance wavelength. The refractive index change may be caused by the presence of cells or by the specific binding of molecules to the surface. Unlike ring resonators and other waveguide based photonic structures [8], [9], photonic crystal resonant surfaces are illuminated with out-of-plane light which gives them the unique advantage of easy integration with a standard microscope. Using hyperspectral imaging of the surface, we can then monitor the resonance wavelength of each pixel in the field of view, enabling the imaging of surface binding events by combining spatial information with sensing information. This combination has added a powerful new imaging modality to the biophotonics toolkit; for example, it allows high-throughput DNA-binding and gene-expression assays [1], it enables determination of cellular attachment to a surface [2], and indication of cell behaviour with respect to a given treatment [4]; it has been shown to enhance fluorescence imaging efficiency [6], [10]; and it could enable the imaging of cellular secretion of specific molecules without the use of fluorescent labels. In addition, it has been used to image single nanoparticles of radius a small as 100 nm [3], [7].

The two key parameters defining this imaging modality are sensitivity and spatial resolution, which is closely related to resonance linewidth. The sensitivity has been explored in detail by Cunningham *et al*. [11], [12], and the spatial resolution and linewidth investigated thoroughly by Block *et al*. [13]. However, the dependence of spatial resolution on refractive index contrast has not been studied yet, and the impact of polarization has not been quantified. Here, we focus on measuring the spatial resolution and study how this resolution depends on the refractive index contrast and on the polarization of the light used. Furthermore, previous studies were conducted on one-dimensionally periodic structures; we explore the performance of 2-D structures as well. Our approach is to deposit a resolution test pattern in resist onto a resonant surface, which allows us to vary the index contrast by adjusting the background index. Since photonic crystal biosensors image refractive index differences, it is crucial to understand their operation as the contrast approaches zero. Indeed, when imaging, for example, living cells in culture media, the refractive index contrast is typically small: on the order 10^−2^ [14].

## 2. Experimental Details

**Fig. 1:**
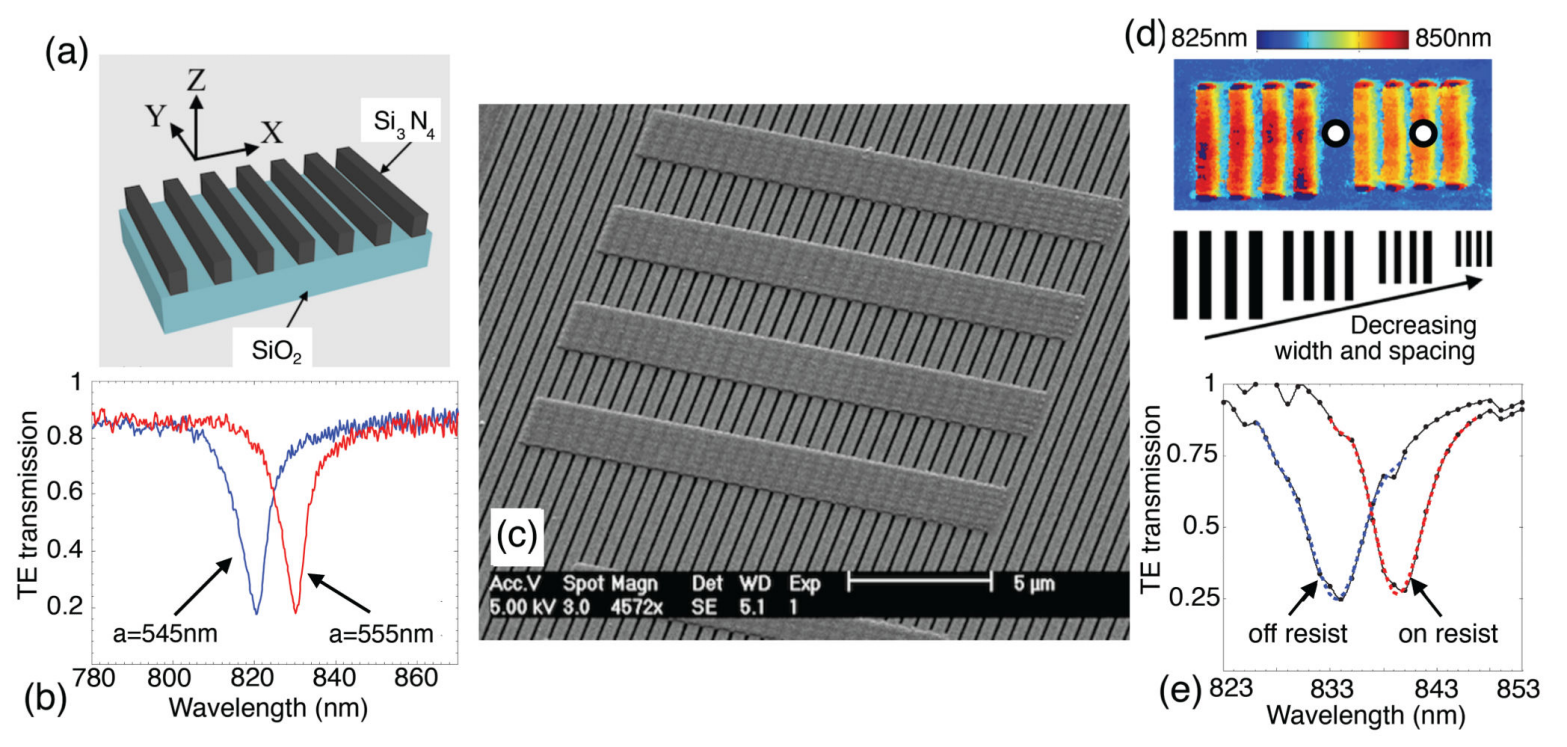
(a) Grating structure. (b) Transmission spectra of gratings with slightly different periods. (c) SEM image of a group of four blocks of FOx resist on top of a silicon nitride grating. (d) Resonance image of two groups of four blocks. The color scale indicates the resonance wavelength in nanometers. (Bottom) Illustration of decreasing the width and the spacing of the groups of resist blocks used to determine the spatial resolution for Fig. 2. (e) Measured spectra from regions indicated by the white circles in (d), showing measured data points (black markers), and fitted Gaussian curves (blue and red dashed lines).

To fabricate a resonant surface, a slab of high refractive index dielectric is structured to form a grating waveguide layer that supports a guided mode resonance (GMR) [15], [16], as shown in Fig. 1(a). For a specific incidence angle, wavelength, grating fill factor, period, thickness, polarization and refractive index, the grating supports a resonant mode excited by diffraction. This resonance manifests itself as a dip or peak in the transmission or reflection spectra, with the thickness of the grating layer being the main parameter which controls whether the device acts as a bandpass or a bandstop filter (the spectra in Fig. 1(b) show the device working as a bandstop filter in transmission). A change of refractive index in the vicinity of the grating shifts the resonance wavelength, which provides the information used to create the final image.

Our resonant surfaces are designed using rigorous coupled wave analysis [17], [18], and fabricated in a 150 nm thick silicon nitride (Si_3_N_4_) layer on a silica substrate. We employ electron beam lithography to pattern a resist layer (Allresist AR-P) after deposition of a ~20 nm layer of aluminium for charge dissipation during e-beam exposure. We follow this with reactive ion etching to transfer the pattern into the dielectric material, using a blend of CHF_3_ and O_2_ gases. We have fabricated both gratings and square arrays of holes (1-D and 2-D gratings, respectively) that display resonances of very similar linewidth at a wavelength of around 830 nm. This wavelength was chosen as it lies in the low-loss therapeutic window of tissue, and because high performance cameras are readily available. The gratings have a period a = 555 nm and a fill factor FF = 80%, and are illuminated with TE polarization (electric field vector pointing along the grating grooves). The hole arrays have a period of a = 540 nm and a hole radius of r = 0.204 a. The resonance wavelength can be finely tuned by varying the period of the structure, as illustrated in Fig. 1(b).

A resonance image is formed by taking a sequence of brightfield images, each at a different illumination wavelength which is achieved by illumination through a tuneable filter (hyperspectral imaging). The resulting hyperspectral cube contains the spectrum from every pixel in the field of view. By analysing the intensity values of each pixel, the resonance wavelength for each pixel can be determined. We fit a curve (a Gaussian or a polynomial) to the measured data from each pixel to accurately obtain the resonance wavelength. Plotting the resonance wavelength of each pixel in the array then gives the resonance image. We use a simple LED (Thorlabs M850L3, 850 nm, 1000 mA) as the illumination source and pass it through a narrow bandpass filter (Semrock LL01-852, 852 nm, ~5 nm FWHM), which is rotated to select the wavelength. The image acquisition (using a Photometrics CoolSnap MYO camera) and filter rotation are controlled by LabView and the image processing is done using MatLab. The use of a bandpass filter allows hyperspectral imaging without the need for an expensive imaging spectrometer or dispersive optics. Due to the relatively large FWHM of the filter (in comparison to the resonance width), we expected to see some broadening of narrow resonances upon measurement, which is the case. However, by choosing a linewidth larger than the filter bandwidth (~10 nm in our case), and due to our curve fitting method, we are able to resolve the resonances used in this paper, and to resolve resonance shifts down to ~0.5 nm. As the grating resonance is sensitive to the refractive index at the surface, an assessment of the spatial resolution can be performed by placing a physical resolution test pattern onto the surface. For comparison, in [13], such a pattern was etched into the grating to produce a local resonance shift. The two techniques appear similar at first sight, but deposition allows us to study the refractive index dependence of the spatial resolution, while etching does not. We spin-coated an electron-beam resist layer (FOx-15, Dow Corning) and exposed groups of blocks of varying size, period and orientation on top of the grating as shown in Fig. 1(c). We discerned from our SEM images that the resist did not travel along the grooves, and that there was some penetration into the grooves, though it is difficult to quantify this. Fig. 1(d) shows a resonance image resulting from the resist bars, and Fig. 1(e) shows measured spectra from the regions indicated by the white circles in Fig. 1(d). The thickness of the resist layer was measured to be approximately 100 nm, and this was kept constant across all our experiments.

## 3. Spatial Resolution Measurements

Firstly, we consider the mechanism that determines the spatial resolution. Ultimately, the spatial resolution depends on the propagation length of the resonant mode inside the grating. The propagation length also determines the resonance linewidth; the more grating periods the mode experiences before being coupled back out, the narrower its spectral linewidth, as is evident from Bragg theory and was already discussed in this specific context in [13]. The propagation length is controlled by the refractive index contrast between the grating ridges and grooves. Therefore, gratings having different spatial resolutions will also have different resonance linewidths and *vice versa*. We also note that the wavelength of light used also affects the achievable spatial resolution (in both x and y directions). In this study, we keep resonance wavelength approximately constant to enable us to focus on the effect of index contrast between objects on the grating surface.

**Fig. 2:**
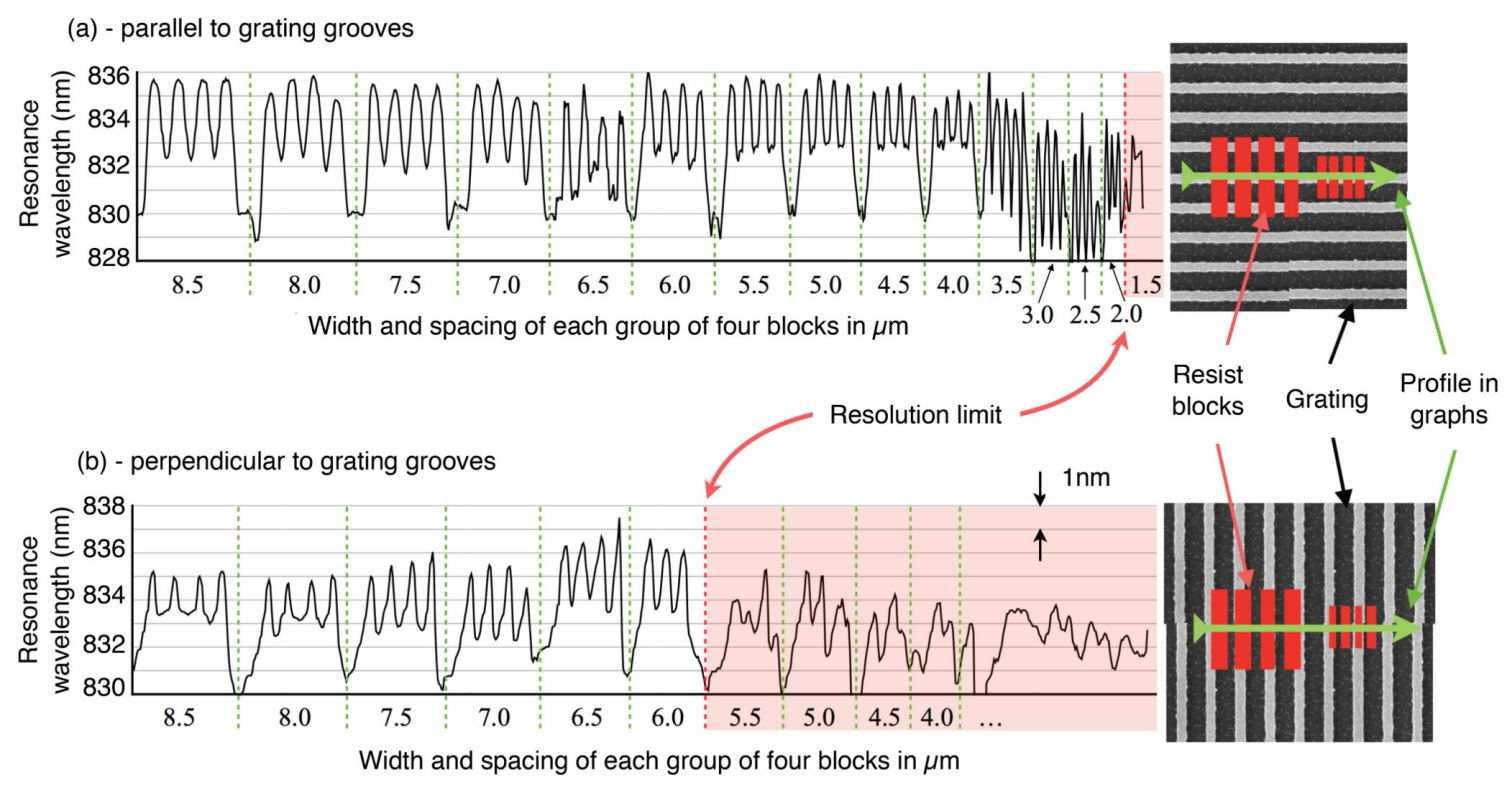
Resonance wavelength profiles from resonance images of groups of four blocks of resist on top of a grating. The width and spacing of each group of blocks is on the horizontal axes in microns. The illustrations on the right show the measurement direction (in green) with respect to the groups of resist blocks (red) and the grating orientation. In (a), the measurement is performed parallel to the grating grooves. In (b), it is perpendicular to the grating grooves. Our limiting resolution, indicated by the red dashed lines, is where the difference in resonance wavelength on and off the individual blocks drops below 1/*e* of the bulk difference between each group of blocks.

**Fig. 3:**
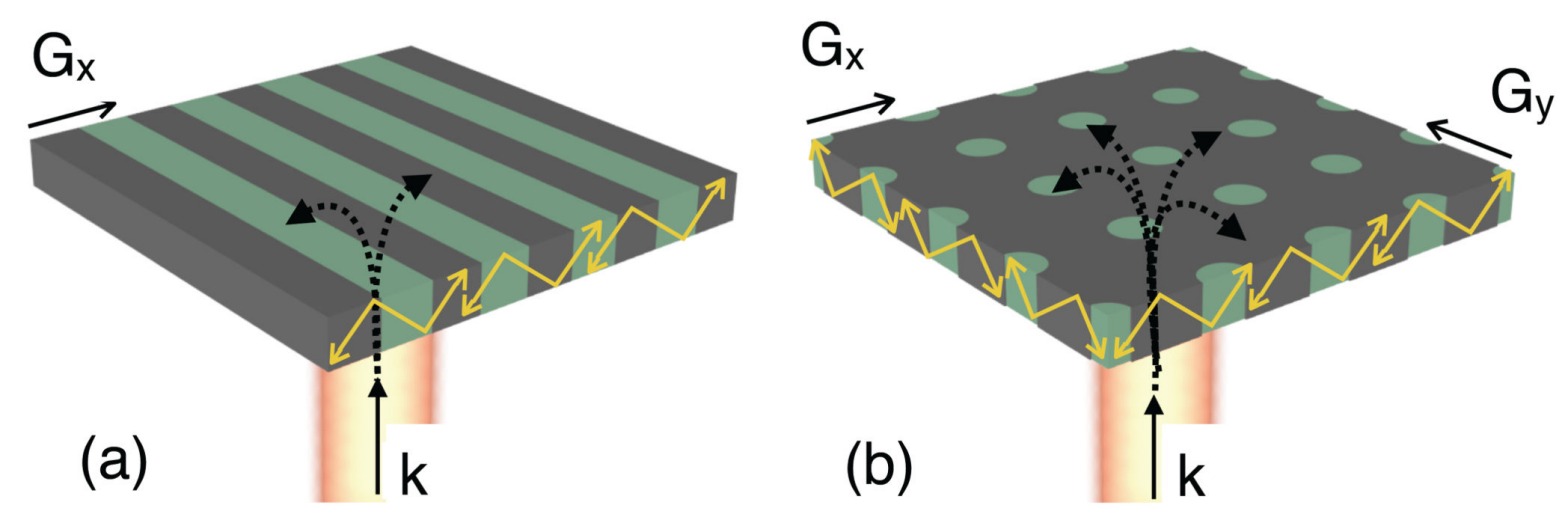
Resonant (a) 1-D and (b) 2-D surfaces. The grating vector (G) allows coupling of incident light with k-vector (k) into resonant modes (shown in yellow) of the grating layer.

Experimentally (see Fig. 2), using our approach of depositing a resist pattern on the surface, we find that the resolution in the direction perpendicular to a 1-D grating [along x in Fig. 1(a)] is 6.0 *μ*m ± 0.5 *μ*m while the resolution parallel to the grating [along y in Fig. 1(a)] is 2.0 *μ*m ± 0.5 *μ*m. A similar asymmetry was previously observed in [13] for an etched-in resolution pattern. One might expect that, since the grating resonance is essential for providing the resulting image, the resolution would be poor along the direction where there is no grating, unlike our observations. This apparent contradiction can be resolved by considering the k-vectors involved, shown in Fig. 3. The incident light only has a k-component in the z-direction, i.e. normal to the grating. The grating vector, which is oriented in the x-direction, then adds a *k_x_* component and light is directed towards the x-direction where it oscillates resonantly. Since there is no *k_y_* component, the light is not directed to the y-direction. Hence the resolution in the y-direction is better, even though the oscillation in the x-direction is required for the resonance to occur in the first place. The resolution in the y-direction is then limited by the imaging system.

**Fig. 4:**
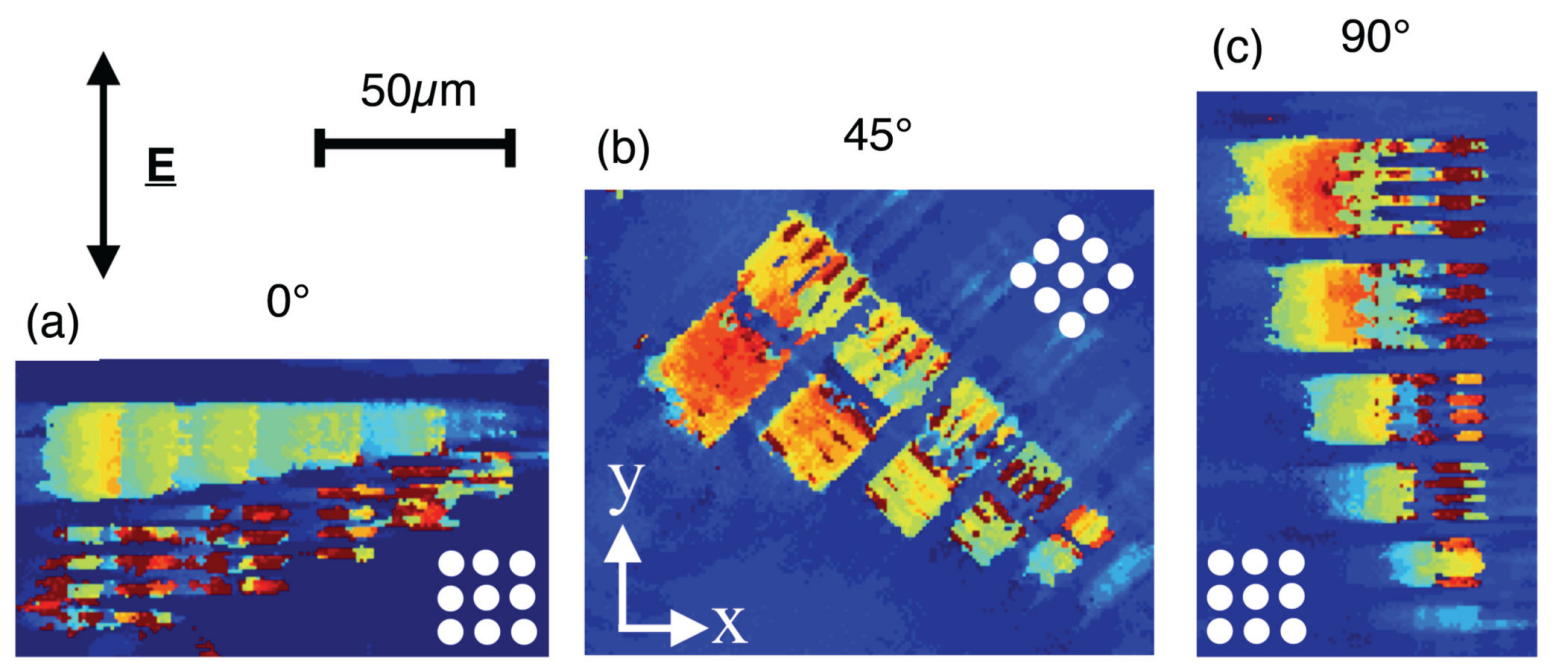
Resonance images of resist blocks deposited on top of a 2-D resonant grating. The incident polarization is in the vertical direction for all cases, as indicated. The sample is rotated from (a) 0° to (b) 45° and (c) 90°, and the orientation of the hole array is indicated in each case. The width and spacing of the five groups of blocks shown is 3.5 *μ*m, 3.0 *μ*m, 2.5 *μ*m, 2.0 *μ*m, and 1.5 *μ*m.

In order to test this hypothesis, we also examined 2-D gratings. If our model is consistent, we would expect that the resolution is symmetric in x and y and that it assumes the lower value (approx. 6.0 *μ*m) determined with the 1-D grating. This expectation assumes that the incident light is unpolarised, thus ensuring that the electric field vector projects on to two equal components along both lattice directions. Since a 2-D grating provides both *k_x_* and *k_y_* components, a resonant mode is excited that oscillates in both directions as in Fig. 3(b). In order to study this, we fabricated square arrays of holes (for details, see above), as a resonant surface with a resonance wavelength and linewidth very similar to that in the 1-D case. Resonance images of resist blocks on the 2-D grating are shown in Fig. 4, where the entire sample has been rotated whilst keeping the incident polarization direction constant. We find equal resolution along both lattice directions for the case where incident polarization is aligned along the diagonal (45°) [see Fig. 4 (b)], in agreement with our model. The 0° and 90° cases (see Fig. 4(a) and (b), respectively) show that resolution is different along the lattice directions, similar to the case of the 1-D grating, even though the grating itself is identical along x and y. We note that resolution is best in the direction of polarization.

The resonant mode we excite is either a TE or a TM waveguide mode which propagates in the plane of the grating, and the excitation of this mode depends on the polarization of the incident light. For unpolarised light, or 45° polarization, the mode is excited equally (and propagates equally) along both lattice directions, resulting in equal resolutions as observed. If, however, the 2-D grating is illuminated with polarised light where E is aligned solely along y, for example [as in Fig. 4(a) or (c)], then diffraction results in a change of the orientation of E associated with the mode propagating in the y direction, while the orientation of E associated with the mode propagating in the x direction is unaffected by diffraction. As a result, the excitation of the guided mode is different in the x and y directions, which leads to a difference in spatial resolution.

## 4. Dependence on Refractive Index Contrast

Since resonant surfaces rely on a refractive index contrast to work at all, it is crucial to understand the limitations of the method as this contrast approaches zero. Our approach of fabricating blocks of resist on top of the resonant surface enabled us to change the refractive index of the surrounding liquid, thereby altering the index contrast between the resist pattern and the background. We used sucrose solutions of varying concentration: a 60%*w/w* solution is close to saturation point (66.7%*w/w* or 2000 g/L) and it yields a refractive index of approximately 1.44 [19]; dilutions with water then produce any value down to 1.33. FOx-15 has a refractive index of 1.39–1.40 [20], however this is reported to increase slightly after electron beam exposure [21]. For the purpose of this paper, we take the index of FOx to be 1.41.

**Fig. 5:**
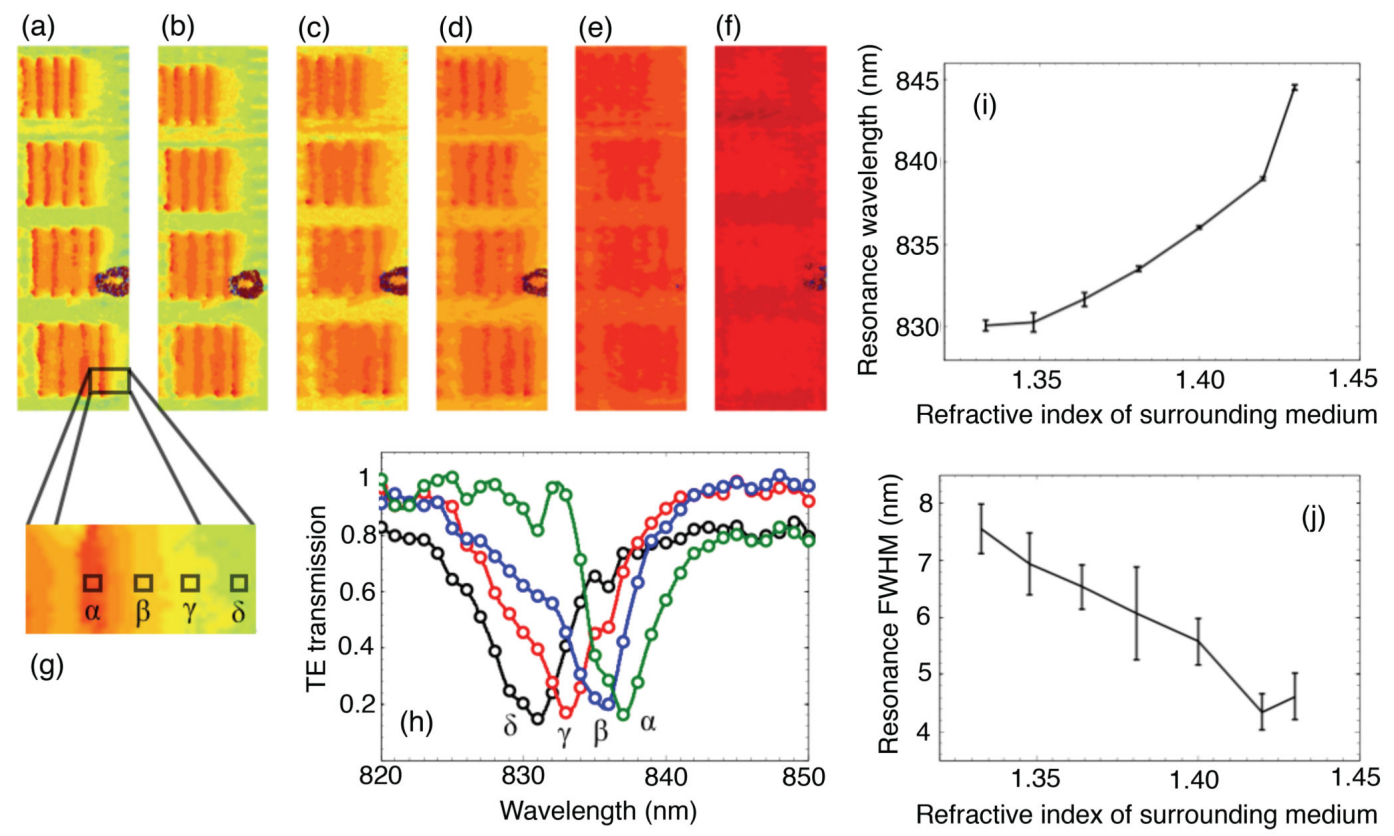
(a)–(f) Resonance images of resist blocks as refractive index contrast is lowered. Δ*n* ≈ 0.077, 0.062, 0.046, 0.029, 0.010, and −0.010 in (a)–(f), respectively. (The negative value indicates that the index of the surrounding liquid is now greater than that of the resist.) The width and the spacing of the bars in each frame is 7 *μ*m for the top group, increasing to 8.5 *μ*m for the bottom group, and the bars are aligned parallel to the grating grooves. Resolution is thus measured perpendicular to the grating grooves, as in Fig. 2(b). (g) Zoomed-in region indicating locations of pixels for the spectra in (h). Average resonance wavelength (i) and FWHM (j) of background pixels vs. surrounding refractive index. Error bars show the standard deviations.

Fig. 5(a)–(f) shows the resonance images of groups of four blocks on top of a 1-D grating as the index of the surrounding liquid is increased. The background becomes red (corresponding to a higher resonance wavelength) because the higher index shifts the resonance upwards in wavelength. The final two images [see Fig. 5(e) and (f)] are taken near Δ*n* = 0, with Fig. 5(f) indicating that we have passed Δ*n* = 0 as the blocks themselves are now lighter than the background (they have a lower resonance wavelength), even though they cannot be individually resolved at this low contrast. We refer to an index contrast that has passed Δ*n* = 0 as being “negative” in this context. Fig. 5(g) indicates the locations of pixels used for the spectra shown in Fig. 5(h), as we move across the edge of a block of resist.

**Fig. 6:**
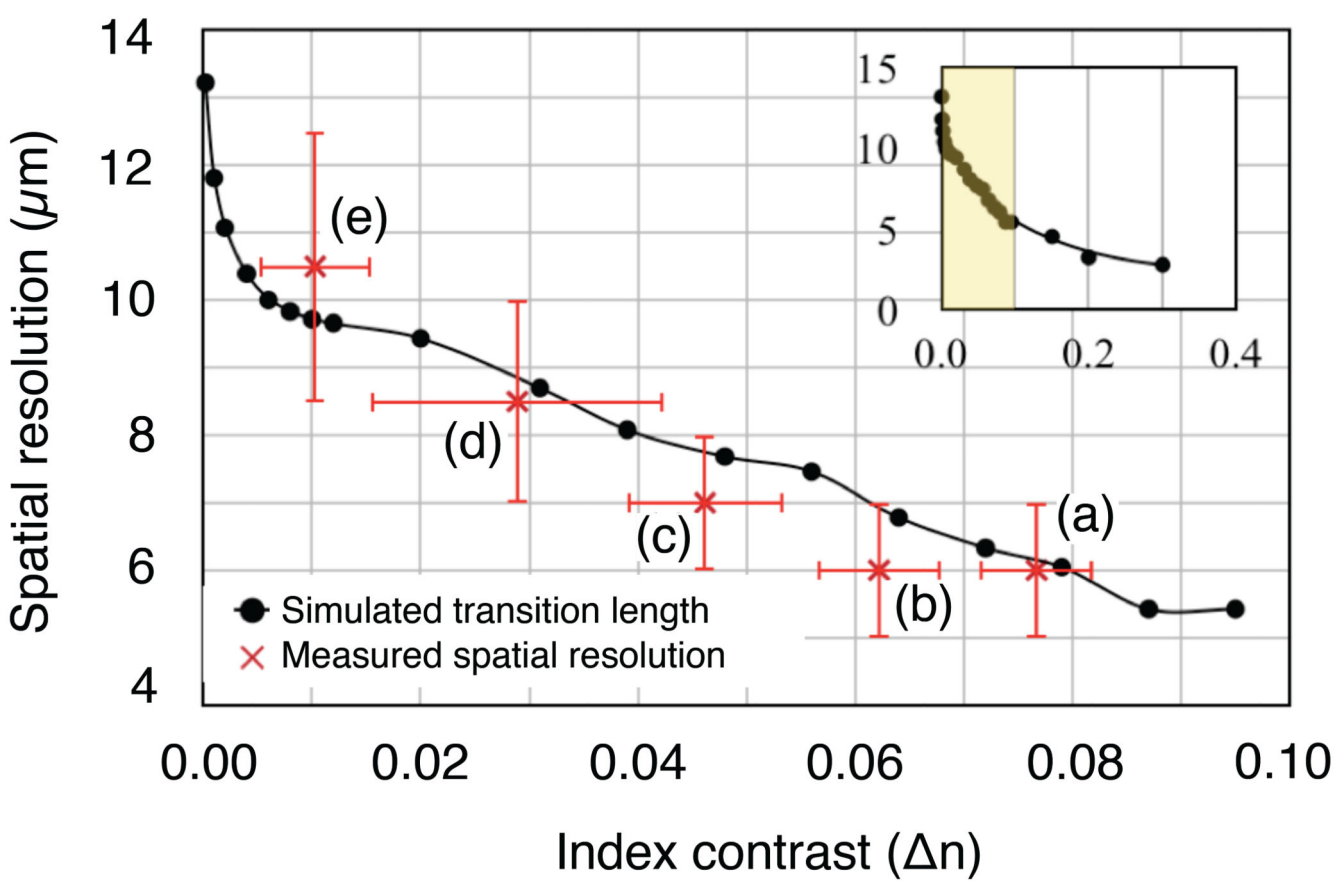
Plot of measured (red) and simulated (black) spatial resolution as a function of refractive index contrast. (Inset) Zoomed out plot of simulated spatial resolution up to higher Δ*n*. The yellow shaded region indicates the area used for the main plot. Letters correspond to the images in Fig. 5(a)–(f).

Fig. 5(i) and (j) shows the resonance wavelength and linewidth determined from a large number of pixels away from any resist blocks—i.e., the background region. As expected, the resonance wavelength increases with refractive index, but it is non-linear. This effect may be explained by the fact that the guided mode is less confined in the silicon nitride layer, and thus has a larger overlap with the surrounding medium. We therefore cannot quote a single value of sensitivity in nm/RIU across the whole measurement range, but our average sensitivity is 150 nm/RIU. The resonance linewidth clearly decreases since the contrast between the silicon nitride and the surrounding medium is lower. As discussed above, this reduction in linewidth is a direct consequence of longer mode propagation in the structure, the same reason for a reduction in spatial resolution discussed below. The measured spatial resolution perpendicular to the grating grooves is plotted in Fig. 6, (red markers), along with the simulated resolution which is discussed below. The resolution was determined in the same way as in Fig. 2(b), i.e., perpendicular to the grating grooves, and shows that the spatial resolution becomes poor at low refractive index contrast. We note here that close to Δ*n* = 0, the refractive index on top of the whole grating is approximately 1.41. This means the propagation length (and therefore spatial resolution) will be larger than for a lower index, such as 1.35. However, the trend shown in Fig. 6 still applies across all sensor surfaces.

**Fig. 7:**
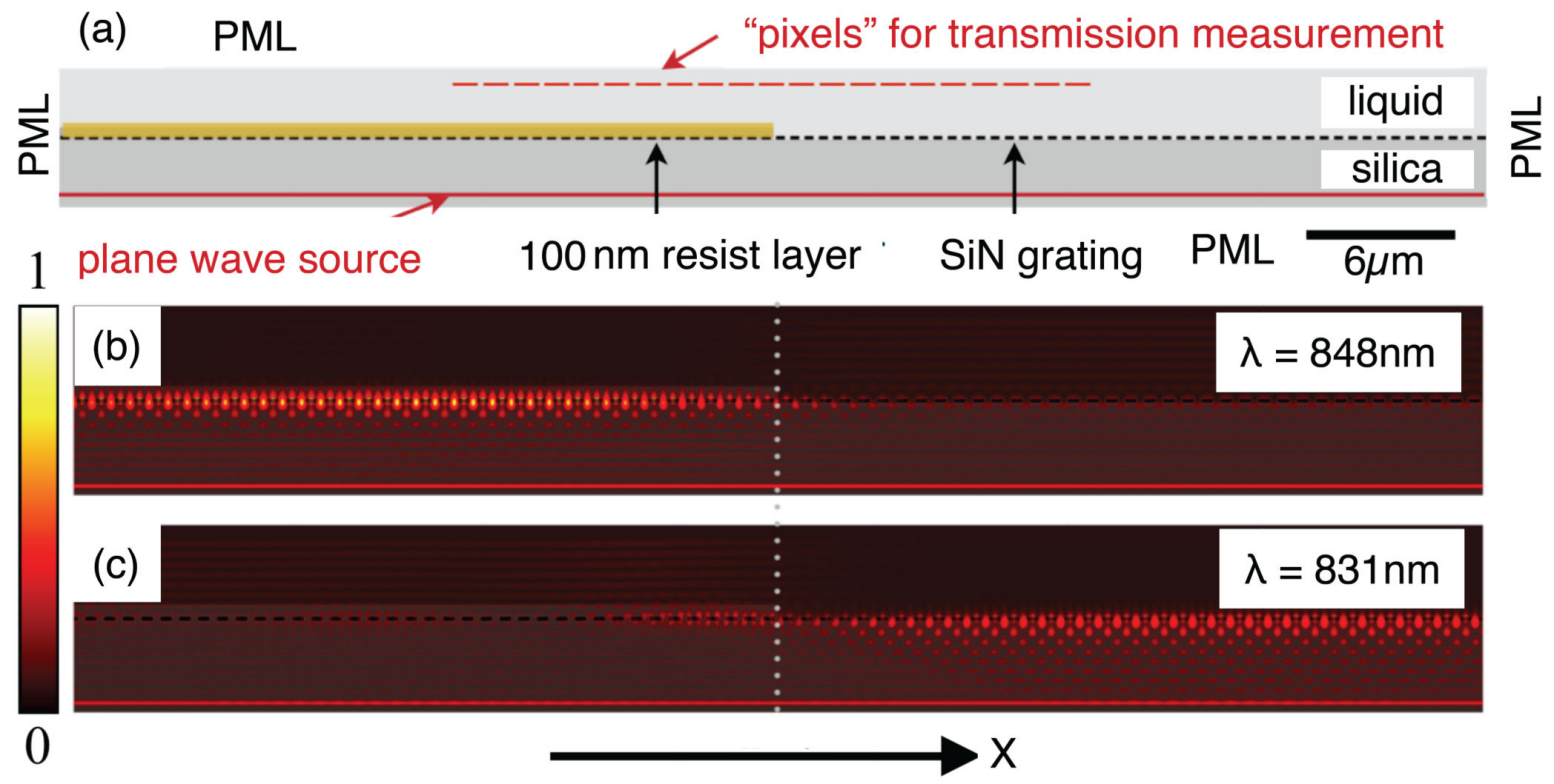
(a) Model used in MEEP simulations. EM field energy density (arb. units) at resonance under the resist (b) and on the liquid-covered grating (c).

**Fig. 8:**
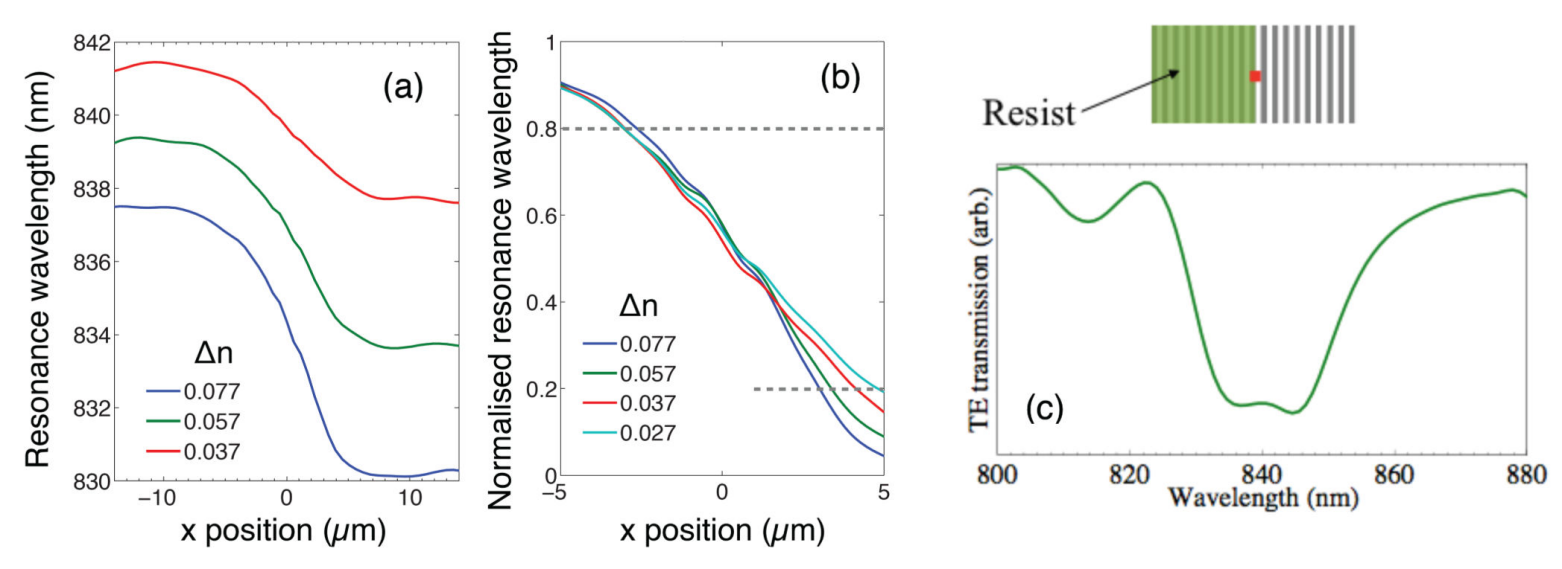
(a) Simulated resonance wavelength against measurement position along x, for four different values of Δ*n*. (b) Normalized (and zoomed-in) version of the curves in (a) to highlight how the slope of the transition depends on Δ*n*. (c) Simulated transmission spectrum from a pixel exactly on the boundary of the resist block, at a contrast of 0.095.

To investigate our results further, we conducted FDTD simulations using MEEP [22]. The model used is illustrated in Fig. 7(a), and consists of a silicon nitride grating of 150 periods, on a glass substrate. A single 100 nm-thick block of resist is placed on top of the left half of the grating, and is set to penetrate into the grooves by 50%, as there was evidence of some penetration into the grooves from our SEM images. The refractive index of the block was set to 1.41 to simulate a layer of FOx resist as used in our experiments, while the index of the surrounding liquid was changed in order to vary the index contrast between the liquid and the resist. We monitored the resonance wavelength as we moved from left to right across the boundary, obtaining the transmission spectrum at each pixel. Fig. 7(b) shows the EM field intensity at resonance on the resist-covered region (848 nm), while Fig. 7(c) is at resonance on the liquid-covered region (831 nm). The resonance wavelength transitions between these two values as we move across the edge of the resist, as shown in Fig. 8(a) for four different refractive index contrasts. We also normalized these curves [see Fig. 8(b)] in order to facilitate a direct comparison of the sharpness of the transition.

Clearly, a smaller Δ*n* causes the slope of the transition to become less steep, and the edge of the resist is effectively blurred out along the x-direction in the final resonance image. To obtain quantitative data from these simulations, we defined a transition length between the two (normalized) resonant wavelengths as being the length between 0.2 and 0.8 for each curve in Fig. 8(b) (indicated by the dashed lines). This data is plotted with the experimental data in Fig. 6 (black markers), and shows that our simulated spatial resolution decreases as we approach zero index contrast, in good agreement with our experimental measurements. Our simulations allowed us to probe down to significantly lower index contrasts than was possible experimentally (as low as 2e^−4^) where we find the spatial resolution rapidly increasing above 10 *μ*m for Δ*n* < 0.01. We were also able to increase the index contrast up to 0.3, where we see the curve becoming flatter (inset in Fig. 6). We note that although these particular curves (Figs. 6 and 8) apply to our specific resonant surface and optical imaging system, the spatial resolution across the grating clearly depends on refractive index contrast, and the trends seen here would be expected to apply to any guided-mode resonance sensor surface. At the boundary of a refractive index difference, we observe the resonance wavelength changing smoothly [Fig. 5(h)]. However, if this transition occurs on a length scale smaller than the pixel size, the measured spectrum may become a superposition of the two resonances, as shown in Fig. 8(c) (simulation). The shape of this superposition also depends on how large the shift between the two individual resonance is, and their linewidth.

An important factor we have not discussed above is the role of scattering losses and surface roughness which may be present on the grating and resist blocks. Scattering losses would mean that the resonant mode decays before achieving the full propagation length, resulting in shorter overall propagation length (and therefore spatial resolution) as well as spectral broadening. The fact that our resonances are spectrally narrow suggests that scattering is not the dominant effect. If the resist blocks were causing significant scattering, we would expect the area underneath the resonances in Fig. 5(h) to change as we move from the bare grating onto the resist, however, the integrals of these curves vary by less than 5%. This, and since we are able to support narrow modes of very similar linewidth to our RCWA simulations leads us to conclude that scattering does not play a significant role in our results.

It is important to note here that the smallest refractive index difference that can be imaged depends on the spectral linewidth of the illumination source; a narrower source allows one to perform a finer sweep of the wavelength during acquisition of the hyperspectral image. With our current setup, we use a bandpass filter with a FWHM of ~5 nm which, if we consider two spectral peaks to be resolved when the depth of the valley between them is 50%, limits our spectral resolution to ~5 nm. We have improved this significantly by fitting a curve to the measured spectra from each pixel [see Fig. 1(e)], and analytically obtaining the peak value. Improved spectral resolution would simply add more data points to the curve in Fig. 6, and would not change the general shape of the curve. It would, however, allow reliable measurements down to even lower index contrasts, which would be an aim of future work.

## 5. Conclusion and Discussion

We have studied the spatial resolution of a silicon nitride resonant surface grating biosensor by depositing a resist pattern on to the surface. We note that the spatial resolution strongly depends on the grating orientation, which we explain with a model based on the k-vectors. We also study the response of a 2-D grating being illuminated with different orientations of polarization. In particular, we show that the spatial resolution for the 1-D grating is 6.0 *μ*m ± 0.5 *μ*m perpendicular to the grating grooves and 2.0 *μ*m ± 0.5 *μ*m parallel to the grooves. Resolution for the 2-D grating, however, is equal along both x and y for unpolarized light, and the resolution becomes asymmetric, as for the 1-D gratings, if polarized light is used. The approach of placing resist patterns on to the grating allowed us to study the effect of refractive index contrast on spatial resolution, which is particularly relevant for biological applications, where the refractive index contrast is typically small. We observe that the spatial resolution becomes worse as refractive index contrast becomes smaller, and the dependence is non-linear. We also observe a narrowing in resonance linewidth due to the reduced contrast between the grating and the surrounding liquid, resulting from the longer resonant mode propagation responsible for the reduction in resolution. Exactly on the boundary of a refractive index difference, we show that for our device, the resonance wavelength transitions smoothly between two values. However, depending on the propagation length of the resonant mode (i.e. the spatial resolution), the measurement pixel size, resonance linewidth and separation, a superposition of two resonances may be measured at a boundary. Finally, our FDTD simulations agree well with our observations, and allowed us to investigate much lower and higher index contrasts than was possible experimentally. In particular, resolution increases above 10 *μ*m when Δ*n* < 0.01.

In comparison to published literature in the field, we draw attention to [6], where 50 *μ*m spots of DNA monolayers are imaged on a resonant surface. Although the spatial resolution is not quoted, from the smearing at the edges of the spots we estimate the spatial resolution to be in the region of 10–20 *μ*m. Without knowledge of the refractive index of the DNA layer, it is impossible to quantitatively compare this work to our results, but qualitatively we would expect the resolution to be 10 s of *μ*m for a low index contrast such as a DNA monolayer. We also draw attention to [7], where individual TiO_2_ nano particles (radius 500 nm) have been detected on top of a resonant surface. The quoted lateral propagation in [7] is 6 *μ*m in the horizontal direction, again comparable to our results. The above authors also report the detection of smaller gold nanoparticles of radii 75–100 nm, and while these gold particles cause a large index contrast and absorption, it is indicated that the resonant mode propagation extends across a similar distance to that observed for the TiO_2_ particles. Lastly, the value of spatial resolution for a TiO_2_ grating surface, reported in [13], is 3.5 *μ*m perpendicular to the grating grooves - comparable to our resolution reported here. The small difference may be due to use of a higher index dielectric and optimal imaging optics, but direct comparisons cannot be made since the resonance shift is induced by etching the resolution pattern into the grating itself, instead of depositing a refractive index object on to the surface. We point out that optimal sensor performance was not a principal aim of this study, and although the data values reported here apply to our specific structure, resonance wavelength, imaging system, choice of resonant mode, and choice of dielectric material, our results are generic to all GMR-based photonic crystal resonant surfaces, in particular regarding their behavior at low index contrast.

## Supplementary Material

fig1_data

fig2_data

fig4_data

fig5_data

fig6_data

fig7_data

fig8_data

refractive_index_data

sup_data_explanation
