# Supplementary figures and images for: Spatial Resolution and Refractive Index Contrast of Resonant Photonic Crystal Surfaces for Biosensing
Source: IEEE Photonics J. Author manuscript; Available in PMC 2015 Sep 7. (PMC4561521; doi:10.1109/JPHOT.2015.2435699)

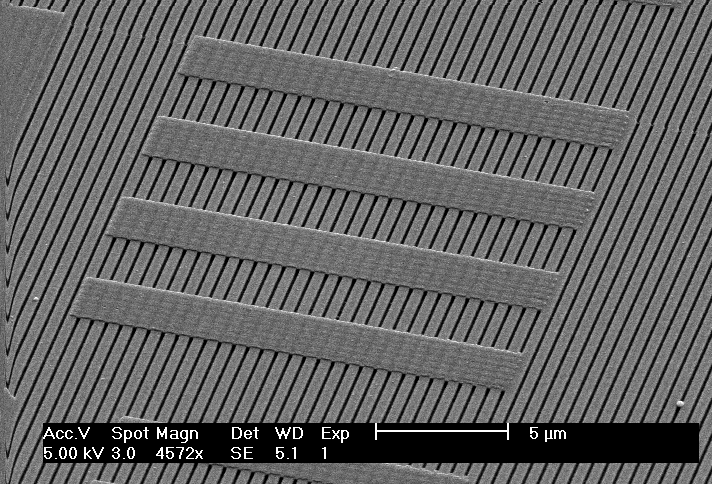

Supplement: fig1_data [file NIHMS64775-supplement-fig1_data.gz › Volumes/graham/MacBook_backup/PhD_folder_2015_06_02/paper_improvements/IEEE_J_Photonics/final_files/data_files/fig1/fig1c.TIF]

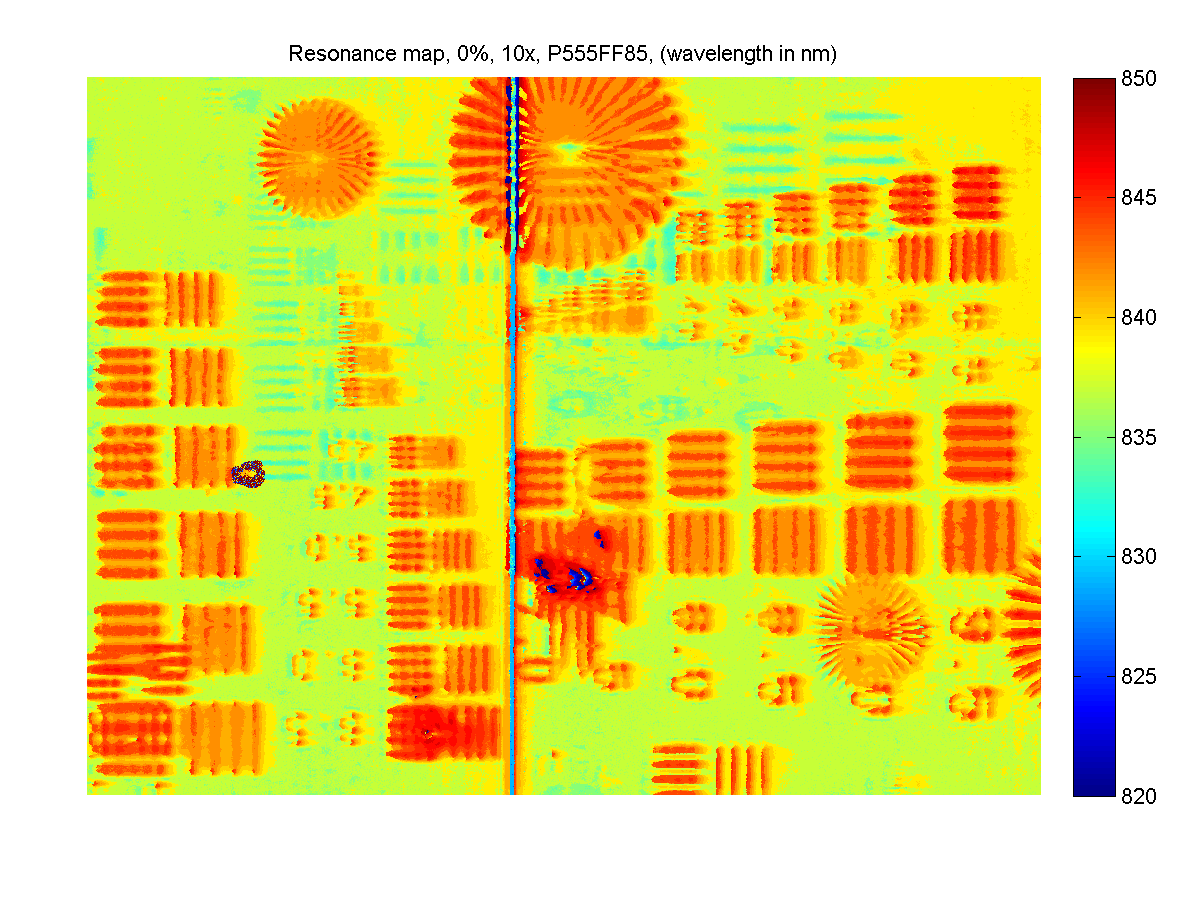

Supplement: fig2_data [file NIHMS64775-supplement-fig2_data.gz › Volumes/graham/MacBook_backup/PhD_folder_2015_06_02/paper_improvements/IEEE_J_Photonics/final_files/data_files/fig2/0pc_p555f85/mapC.png]

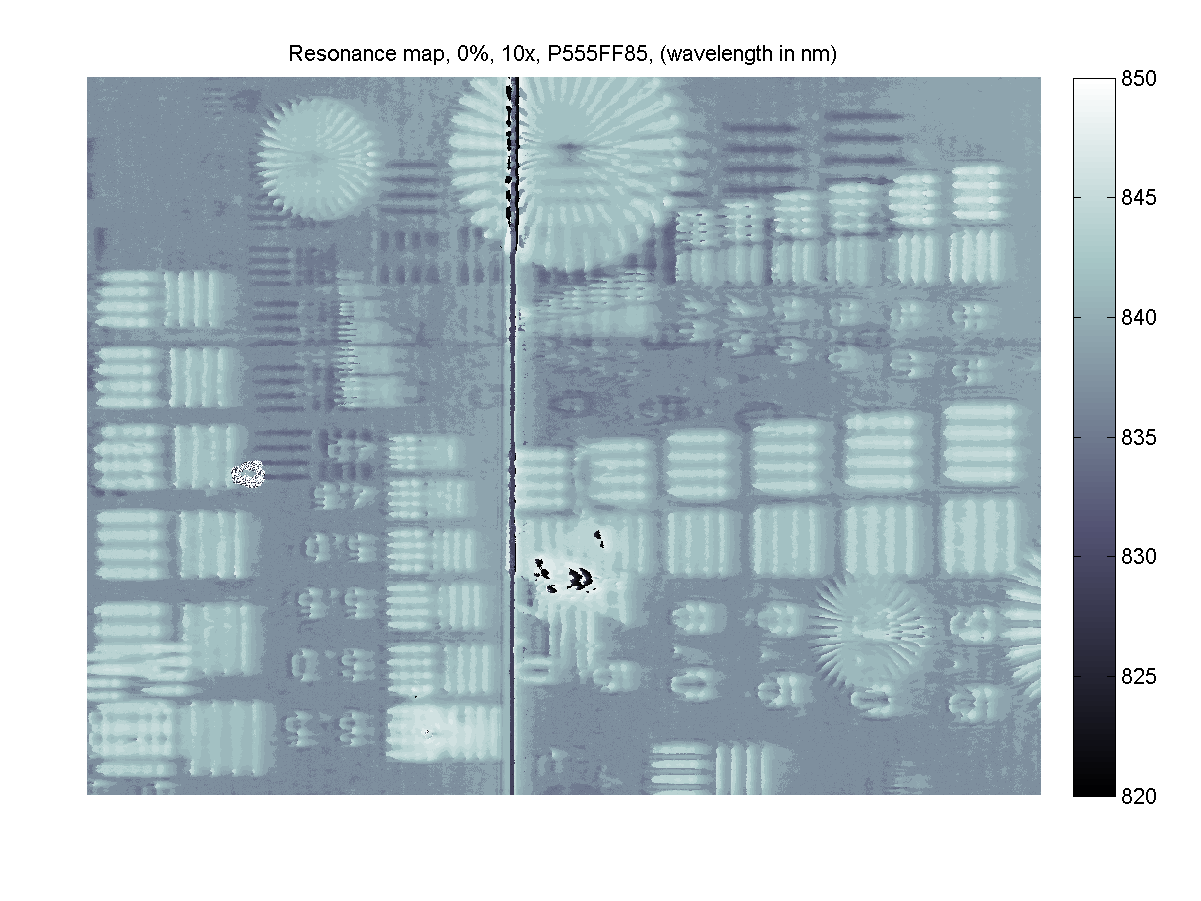

Supplement: fig2_data [file NIHMS64775-supplement-fig2_data.gz › Volumes/graham/MacBook_backup/PhD_folder_2015_06_02/paper_improvements/IEEE_J_Photonics/final_files/data_files/fig2/0pc_p555f85/mapG.png]

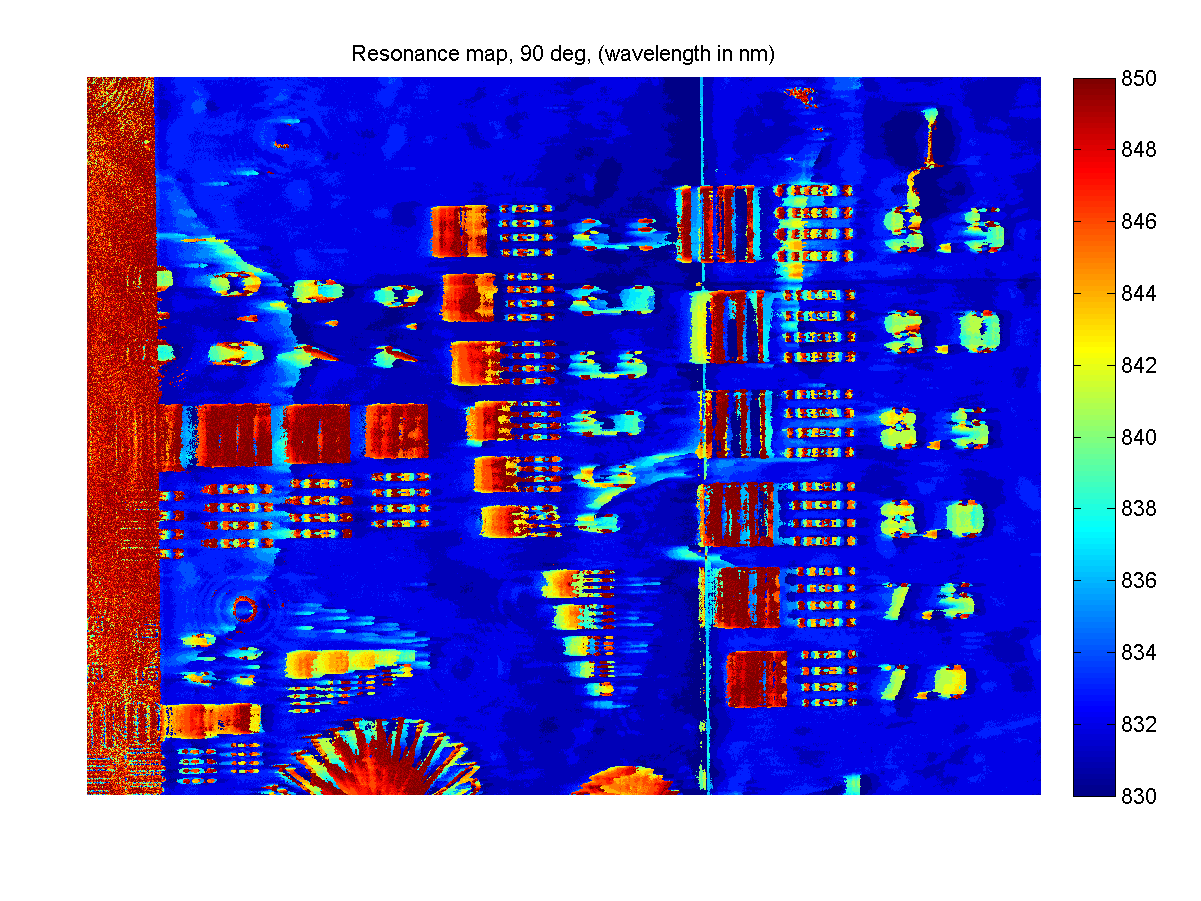

Supplement: fig4_data [file NIHMS64775-supplement-fig4_data.gz › Volumes/graham/MacBook_backup/PhD_folder_2015_06_02/paper_improvements/IEEE_J_Photonics/final_files/data_files/fig4/4c_raw/90mapC2.png]

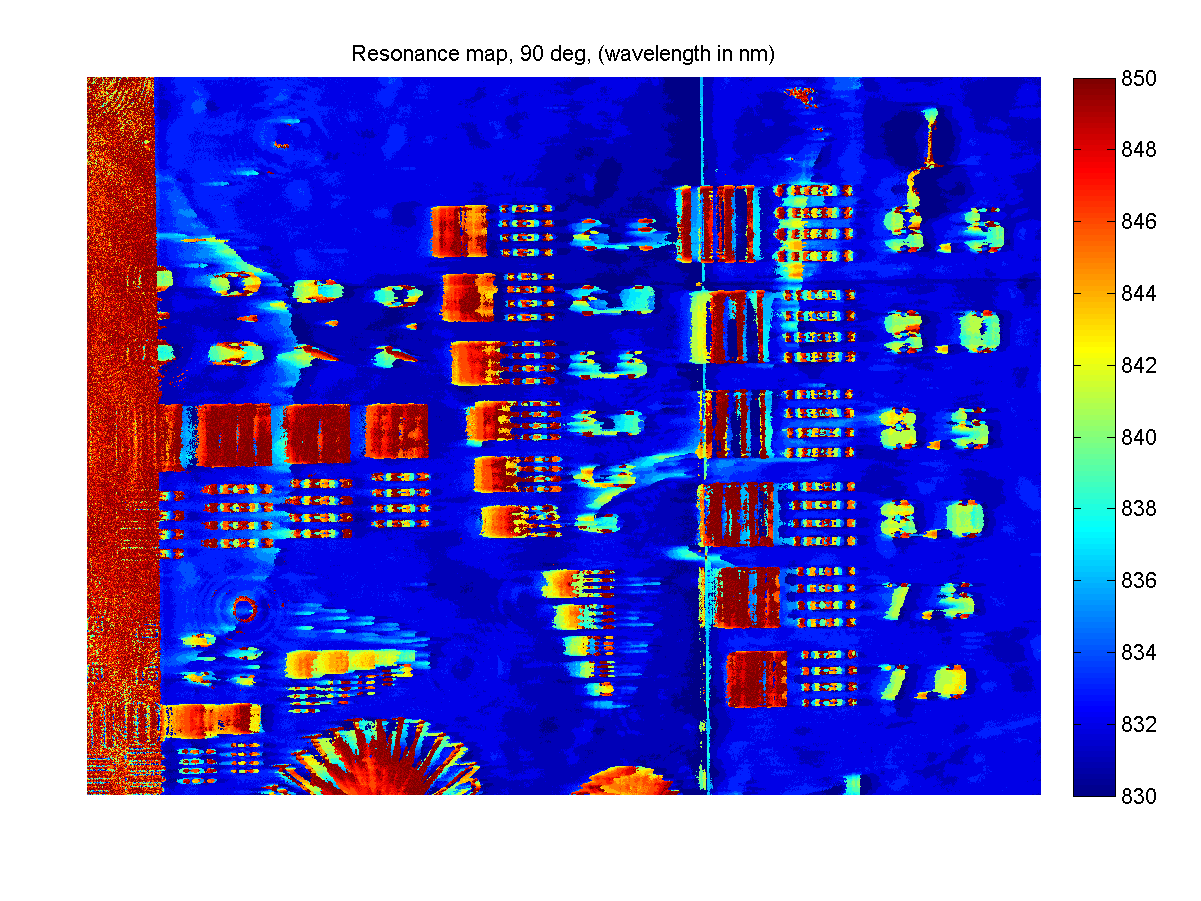

Supplement: fig4_data [file NIHMS64775-supplement-fig4_data.gz › Volumes/graham/MacBook_backup/PhD_folder_2015_06_02/paper_improvements/IEEE_J_Photonics/final_files/data_files/fig4/4c_raw/mapG2.png]

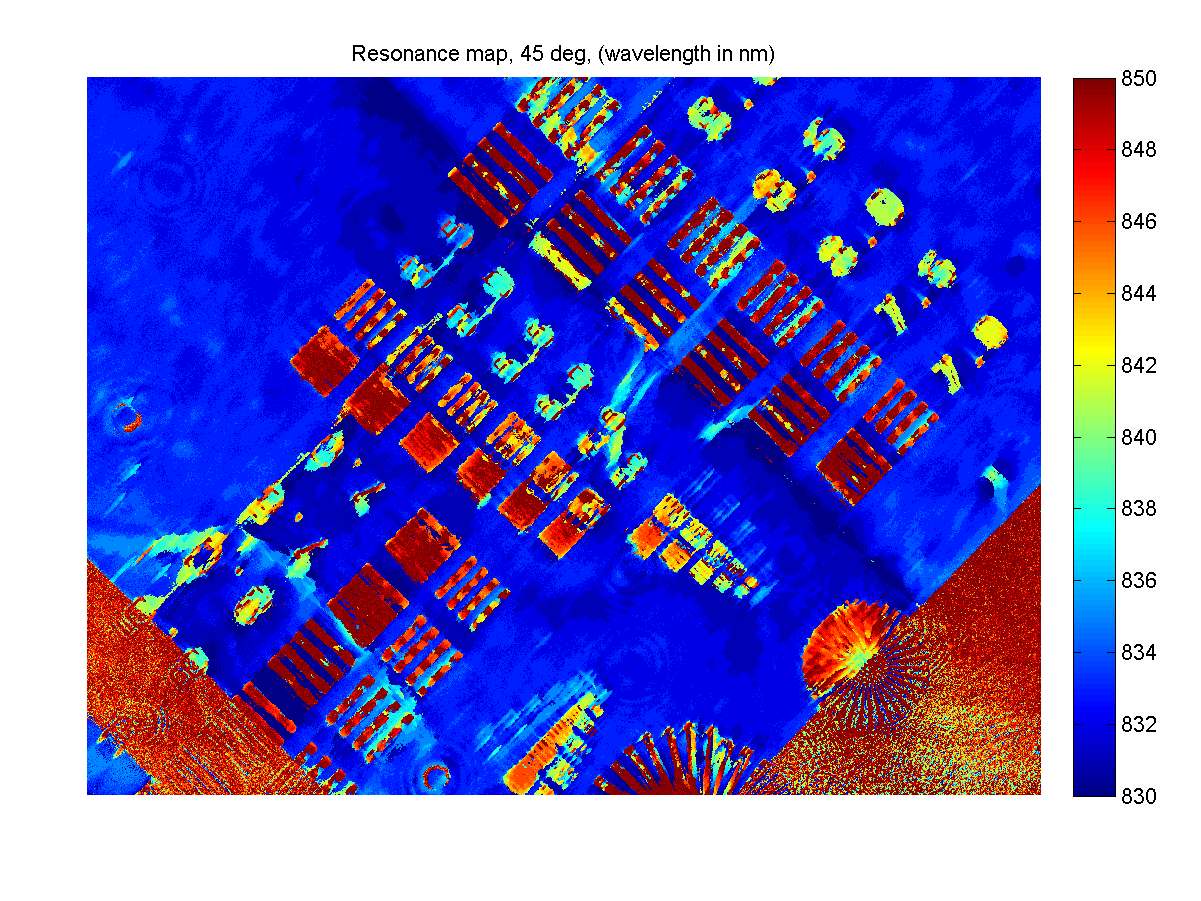

Supplement: fig4_data [file NIHMS64775-supplement-fig4_data.gz › Volumes/graham/MacBook_backup/PhD_folder_2015_06_02/paper_improvements/IEEE_J_Photonics/final_files/data_files/fig4/4b_raw/45mapC2.png]

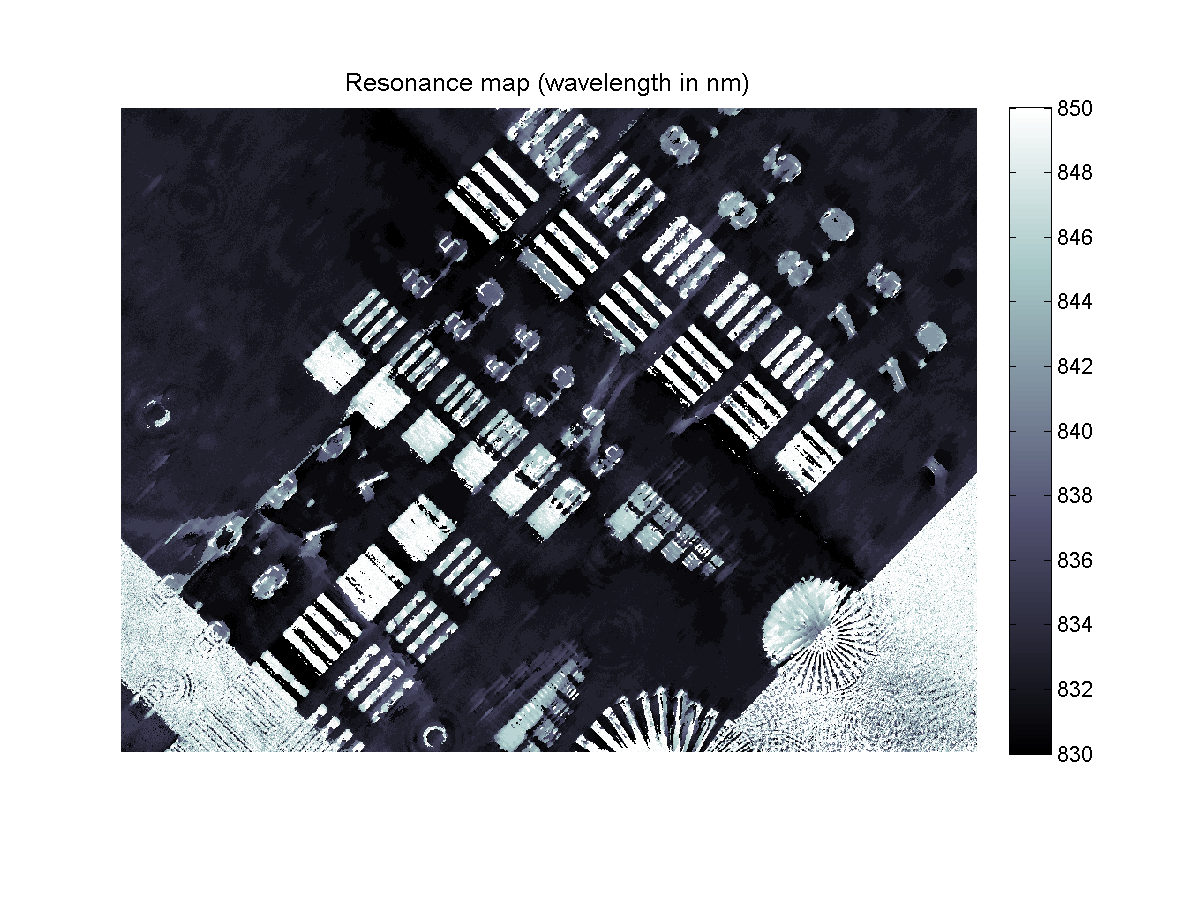

Supplement: fig4_data [file NIHMS64775-supplement-fig4_data.gz › Volumes/graham/MacBook_backup/PhD_folder_2015_06_02/paper_improvements/IEEE_J_Photonics/final_files/data_files/fig4/4b_raw/G45.png]

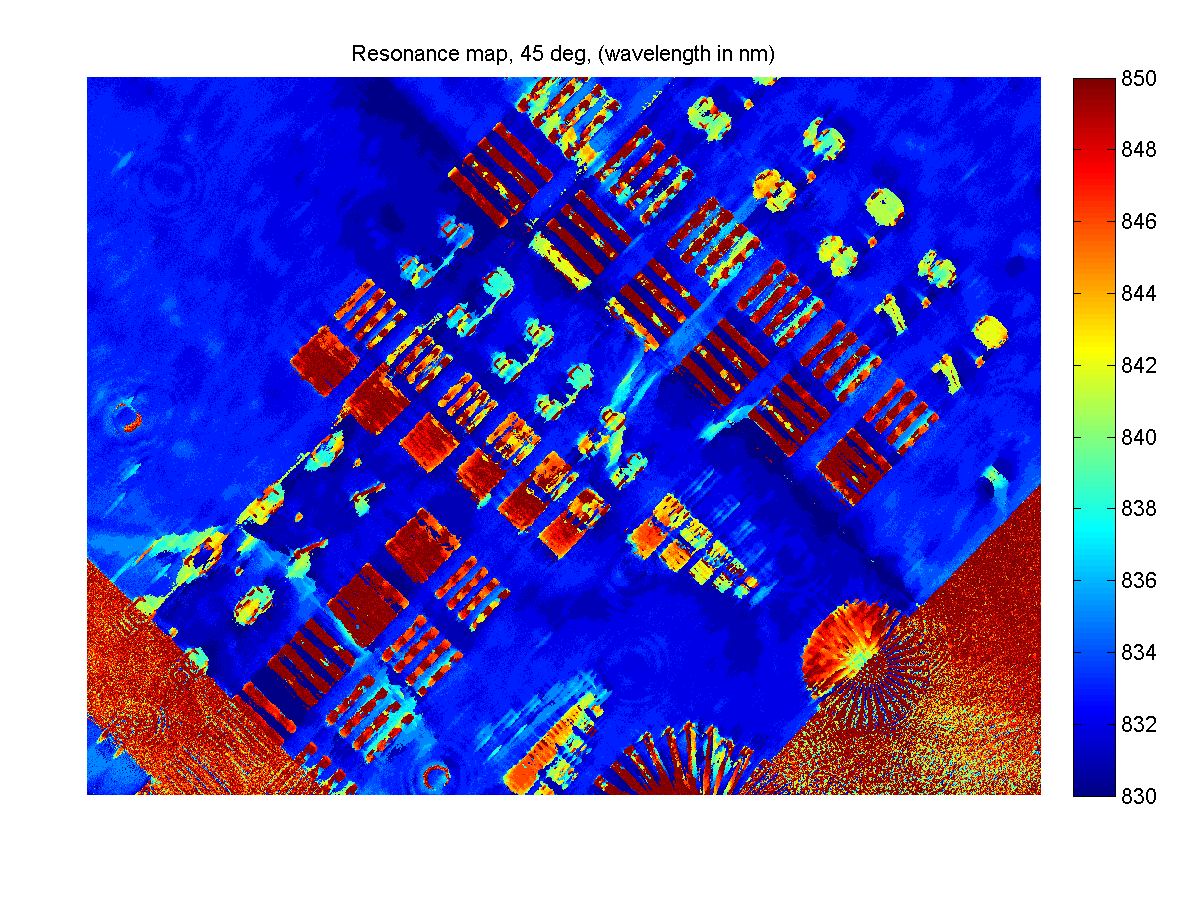

Supplement: fig4_data [file NIHMS64775-supplement-fig4_data.gz › Volumes/graham/MacBook_backup/PhD_folder_2015_06_02/paper_improvements/IEEE_J_Photonics/final_files/data_files/fig4/4b_raw/mapG2.png]

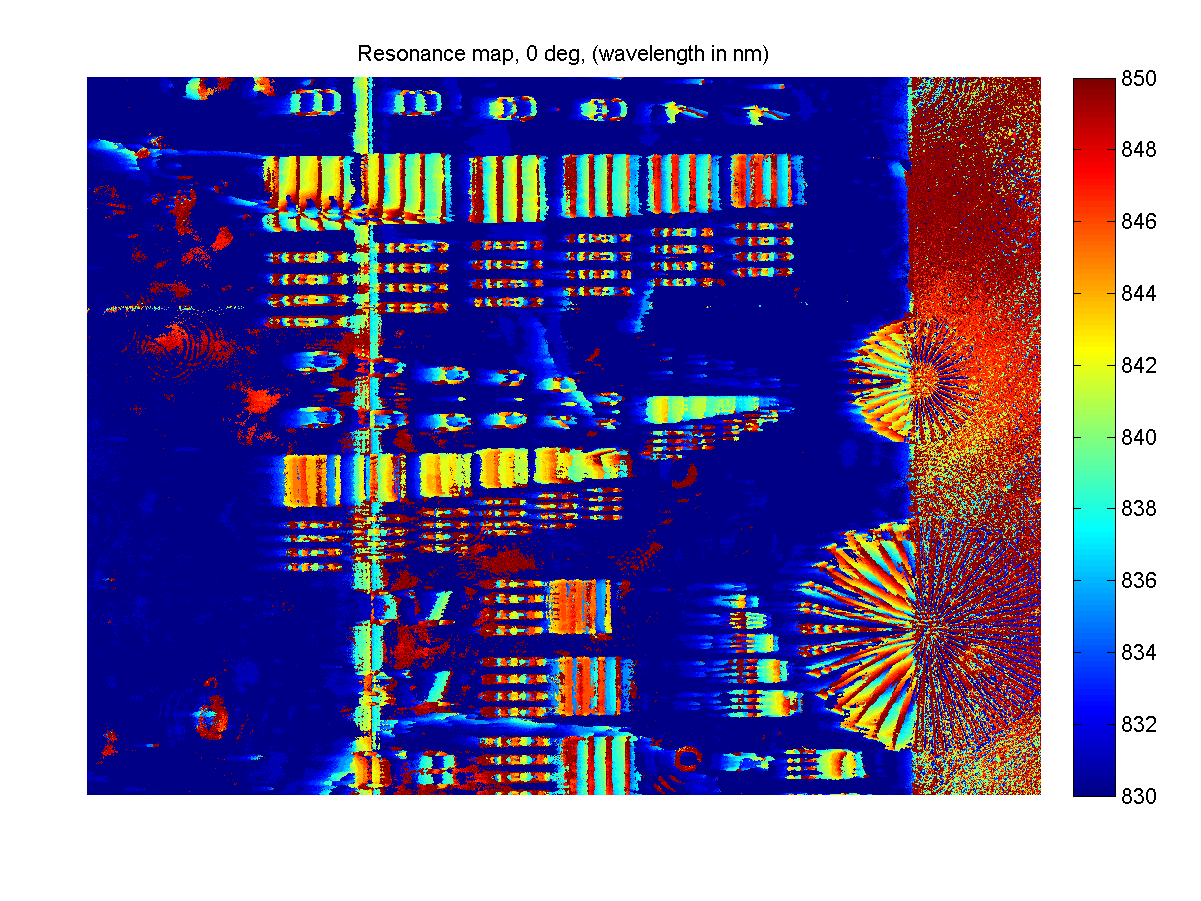

Supplement: fig4_data [file NIHMS64775-supplement-fig4_data.gz › Volumes/graham/MacBook_backup/PhD_folder_2015_06_02/paper_improvements/IEEE_J_Photonics/final_files/data_files/fig4/4a_raw/0mapC2.png]

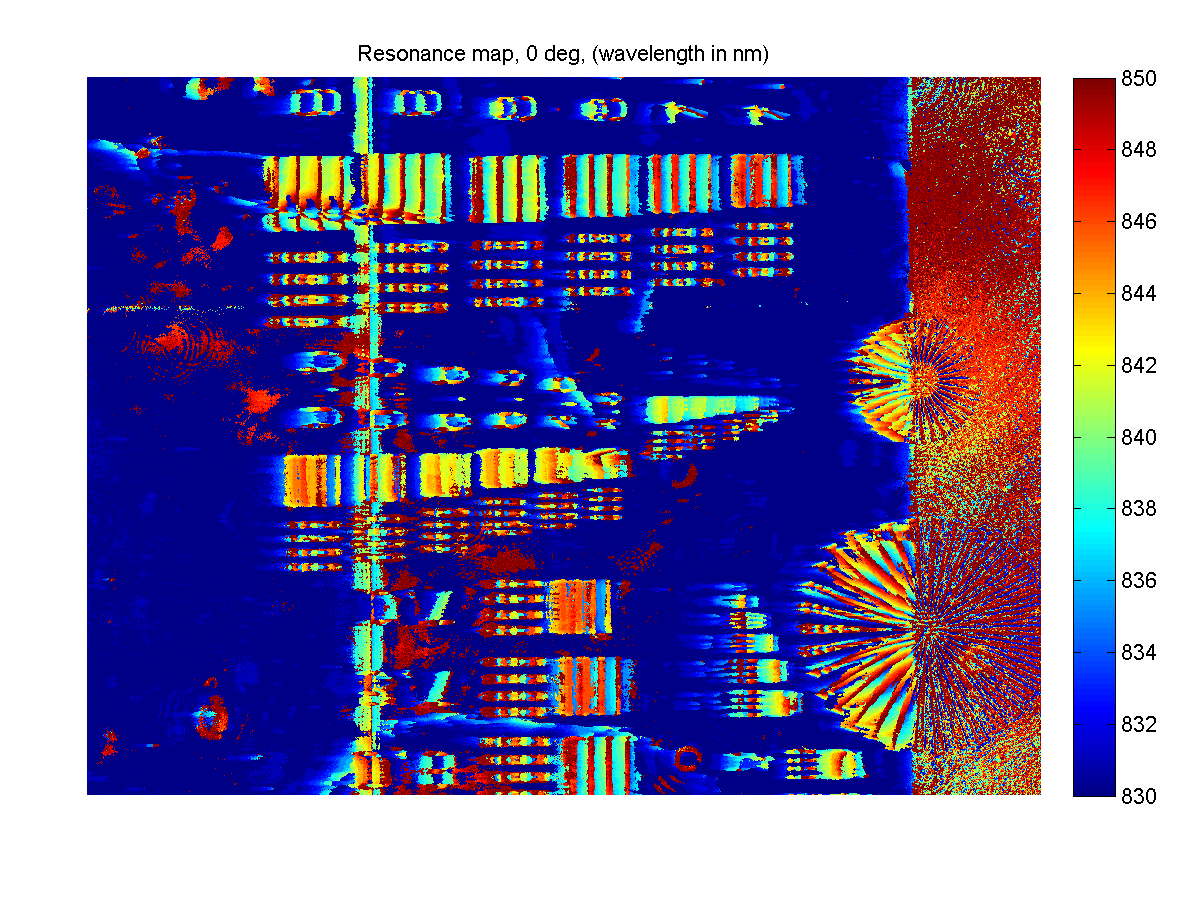

Supplement: fig4_data [file NIHMS64775-supplement-fig4_data.gz › Volumes/graham/MacBook_backup/PhD_folder_2015_06_02/paper_improvements/IEEE_J_Photonics/final_files/data_files/fig4/4a_raw/mapG2.png]

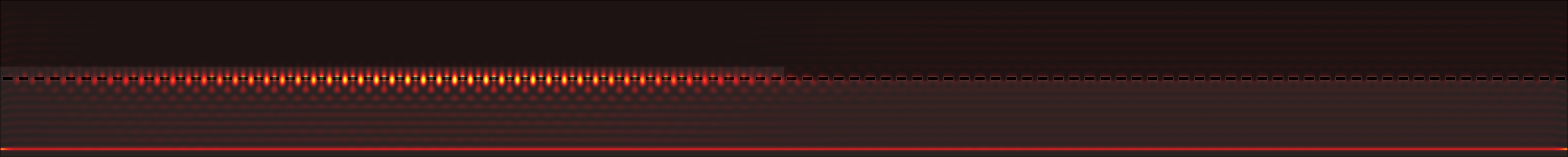

Supplement: fig7_data [file NIHMS64775-supplement-fig7_data.gz › Volumes/graham/MacBook_backup/PhD_folder_2015_06_02/paper_improvements/IEEE_J_Photonics/final_files/data_files/fig7/fig_7b.png]

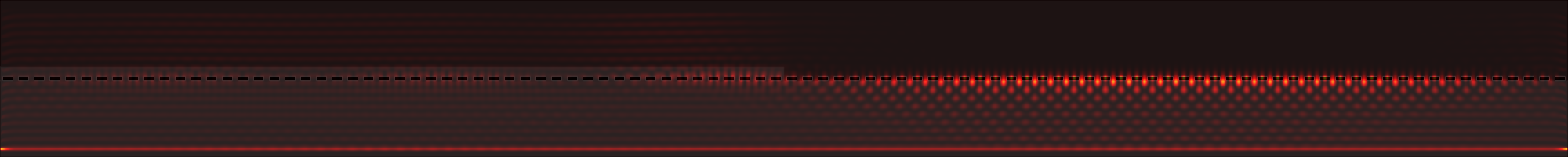

Supplement: fig7_data [file NIHMS64775-supplement-fig7_data.gz › Volumes/graham/MacBook_backup/PhD_folder_2015_06_02/paper_improvements/IEEE_J_Photonics/final_files/data_files/fig7/fig_7c.png]
